# Fear and Challenges of Nursing Students Being in Hospital for Clinical Posting During the COVID-19 Pandemic: An Exploratory Survey

**DOI:** 10.3389/fpsyg.2022.867606

**Published:** 2022-05-09

**Authors:** Nimarta Rana, Nipin Kalal, Suresh K. Sharma

**Affiliations:** College of Nursing, All India Institute of Medical Sciences Jodhpur, Jodhpur, India

**Keywords:** challenges, clinical posting, COVID-19 pandemic, fear, hospital, nursing students

## Abstract

**Background:**

The world is facing unprecedented challenges in the face of a global pandemic (COVID-19). The institutions resumed nursing students’ clinical experiences as an earlier part of their curriculum, which was transitioned to a virtually delivered format due to global disaster. Therefore, working through this pandemic in hospital posting is challenging and fearful for nursing students. The aim of this study was to measure the fear of COVID-19 and the challenges faced by nursing students when posted in hospitals during the COVID-19 pandemic.

**Methods:**

A web-based exploratory survey was conducted on 185 participants from March 2021 to April 2021. Participants were selected through a web-based survey (Google form) by non-probability purposive sampling technique. The Fear of COVID-19 Scale and self-structured questionnaires with the Likert-type scale were used to measure the fear of COVID-19 and the challenges faced by nursing students when posted in hospitals during the COVID-19 pandemic, respectively. Descriptive and inferential statistics were used for the analysis of data with IBM SPSS version 27.

**Results:**

A significant number (61.1%) of participants had moderate fear of COVID-19 infection, one-third of them (28.1%) had mild fear, and very few participants (10.8%) had an extreme fear of COVID-19 infection. The majority of participants (64.9%) faced moderate challenges, 27% faced high challenges, and very few study participants (8.1%) did not face any challenges when posted in the hospital during the COVID-19 pandemic. The fear due to COVID-19 is not associated with demographic variables, but the challenges faced due to COVID-19 are significantly associated with demographic variables, such as the age, batch, and duration of clinical posting (*p*-value = 0.01).

**Conclusion:**

Study data indicated that respondents reported fear of COVID-19 infection and also experienced a variety of challenges in hospital posting during the COVID-19 pandemic.

## Introduction

The nationwide coronavirus (COVID-19) pandemic and the ensuing lockdown have forced schools and institutions across India to temporarily shut down to prevent the spread of the virus. This unprecedented move had created a big gap in the education system despite the central and state governments doing their best to provide support for e-learning and online education. The crisis in the world scenario, caused by COVID-19, has demanded prompt changes in the way of teaching in institutions. Several institutions in the country started online education to help students to continue their education from home (Dewart et al., 2020).

Consequently, the pandemic has elicited significant distractions in the implementation of programs across educational institutions, and the most affected are the students (De Los Santos et al., 2021). The disruption of education for nursing students had been unanticipated by the students (Aslan and Pekince, 2021). In addition, nursing students also retracted from a clinical practicum in March 2020, specifically because of the possibility that a nursing student, who was an asymptomatic or mildly symptomatic carrier of COVID-19, could return to their community and spread the infection further. If nursing students were exposed to COVID-19 in a clinical environment, they put not only their health at risk but also the health of their families (Dewart et al., 2020). Lira et al. (2020) performed a reflection analysis using a theoretical approach in which they explored the challenges and perspectives of nursing education in times of the COVID-19 pandemic. Long-standing problems have arisen as a result of the pandemic, and the processes of acceleration, change, and paralysis have marked education in these times (Lira et al., 2020). De Los Santos et al. (2021) conducted a cross-sectional study on fear of COVID-19, poor quality of sleep, irritability, and intention to quit school among nursing students. According to the results of the report, first-year nursing students are the most fearful of the group.

Fear of COVID-19 is linked to high irritability, poor sleep quality, and a desire to drop out of nursing school. As we all know, the COVID-19 vaccination drive is currently taking place across the world. In addition, the institutions restored nursing students’ clinical experiences as an earlier part of their curriculum, which was transitioned to a virtually delivered format due to the global disaster, the COVID-19 pandemic. Thus, working through this pandemic in hospital clinical posting is challenging and fearful for nursing students because of the fear of contracting the disease and the risk of transmitting it.

Nursing students, without a doubt, become familiar with quickly adjusting, routing through areas, and moving promptly from one challenge to the next. Working through a pandemic has changed all aspects of the general population’s lives as well as of nursing students.

Hence, this study was conducted to assess the fear of COVID-19 and the challenges faced by nursing students being in hospital for clinical posting during the COVID-19 epoch.

## Materials and Methods

### Research Design

A web exploratory survey was conducted to find out the fear and challenges of nursing students being in hospital for clinical posting during the COVID-19 pandemic.

### Setting and Data Collection

The study was conducted among undergraduate nursing students in March 2021 in western Rajasthan, India. The study aimed to measure the fear of COVID-19 and challenges faced by nursing students when posted in hospitals during the COVID-19 pandemic. The survey link was forwarded to participants *via* WhatsApp. The researcher followed up on the status of data collection a week after the survey link was delivered.

### Participants

The sample size was estimated by using Slovin’s formula: *n* = N/(1 + Ne^2^), where n is the number of samples, *N* is the population size (total students: 255), and e is the margin of error of 5%, with 95% CI, and the minimum estimated sample size needed is 154. After considering the withdrawn participants, 20% of preliminary participants, approximately 185 subjects, were enrolled in the study. The students were recruited *via* a non-probability purposive sampling technique following the inclusion criteria, where only undergraduate students who have consented to participate were included in the study. A survey link (Google form) was shared and circulated among participants *via* a WhatsApp group to facilitate data collection. Duplication of data was restricted since one student can submit the response through only one e-mail ID.

### Data Collection Instruments

Three questionnaires section were used to assess the main variables of the study.

### Part I: Demographic Variables

The first questionnaire includes 6 items related to the demographic variables of the respondents: age, batch, religion, history of COVID-19 infection, family history of COVID-19 infection, and duration of clinical posting in the pandemic.

### Part II

The fear of COVID-19 was assessed using the Fear of COVID-19 Scale by Ahorsu et al. (2020). The participants indicated their level of agreement with the statements. It has a list of seven items on a five-item Likert-type scale. Answers included “strongly disagree,” “disagree,” “neutral,” “agree,” and “strongly agree.” The minimum score possible for each question is 1, while the maximum is 5. A total score could be calculated by adding up each item’s score (ranging from 7 to 35). The scale has composite reliability of 0.88, which indicates good reliability in measuring the fear construct.

### Part III

The third section intended to measure the challenges faced by nursing students when posted in hospitals during the COVID-19 pandemic. The tool was prepared after conducting three focussed group discussions (FGD) of 15–20 min with undergraduate students of different batches. This scale was validated by experts and found to be 80% reliable with Cronbach’s α = 0.81. The content validity ratio (CVR) for the scale was 0.89, showing an acceptable level of internal consistency of the scale. It has a list of 10 items on a three-item Likert-type scale. Answers included “always,” “sometimes,” and “never.” The minimum score possible for each question is 1, and the maximum is 3. A total score could be calculated by adding up each item’s score (ranging from 10 to 30). The higher the scores interpreted, the higher the challenges faced by nursing students. The score given for the “always” response will be 3, the “sometimes” response will be 2, and the “never” response will be 1. The total score could be calculated by adding up each score (ranging from 10 to 30).

### Data Analysis

SPSS version 27, IBM Corp., Armonk, NY, United States was used to aid in the analysis of the variables. The data were analyzed using both descriptive and inferential statistics. Frequency and percentage were calculated for demographic variables, fear, and challenges. Mean and standard deviation was considered for the assessment of fear and challenges due to COVID-19 in all domains. The normality of the data was checked with the help of Kolmogorov-Smirnov. It was found that data are normally distributed. An independent *t*-test and ANOVA tests were used to find the association of demographic variables with fear and challenges due to COVID-19 among participants. The data significance was set to less than 0.05.

## Results

Table 1 displays the demographic variables of study participants. A total of 185 participants participated in the study. A majority of the participants [120 (64.9%)] were aged more than 21 years, and one-third of them [65 (35.1%)] were aged less than 20 years. The maximum numbers of participants [96 (51.9%)] were from the Intermediate level (second year and third year) and around half of the study participants [45 (24.3%) and 44 (23.8%)] were freshman (first year) and interns (fourth Year), respectively. In regard to religion, the maximum number of participants were Hindu [181 (97.8%)] and only few participants [4 (2.2%)] were from other religions (Muslim/Sikh). The maximum number of participants [167 (90.3%)] had no history of COVID-19 infection and only few participants [18 (9.7%)] had a history of infection with COVID-19. The maximum number of participants’ [175 (94.6%)] family members had no COVID-19 infection and only a few participants’ [10 (5.4%)] family members had COVID-19 infection. The of study participants, 132 (71.4%), had 2 weeks of clinical posting during the COVID-19 pandemic and less than half of the participants, 53 (28.6%), had 3 weeks of clinical posting during the COVID-19 pandemic.

**TABLE 1:** Frequency and percentage distribution of demographic variables.

Variables	Frequency (%)
**Age**	
≤ 20 Years	65 (35.1)
≥ 21 Years	120 (64.9)
**Batch**	
Fresher (1st Year)	45 (24.3)
Intermediate (2nd and 3rd Year)	96 (51.9)
Interns (4th Year)	44 (23.8)
**Religion**	
Hindu	181 (97.8)
Others (Muslim/Sikh)	04 (2.20)
**Did you ever had history of COVID-19 infection?**	
Yes	18 (9.70)
No	167 (90.3)
**Did any of your family member had COVID-19 infection?**	
Yes	10 (5.4)
No	175 (94.6)
**Duration of clinical posting during COVID-19 pandemic**	
≤ 2 Week	132 (71.40)
≥ 3 Week	53 (28.60)

*N = 185.*

Table 2 depicts the fear of participants related to COVID-19; more than half [113 (61.1%)] of the study participants have a moderate fear of COVID-19 infection, one-third of study participants [52 (28.1%)] have mild fear, and only a few participants [20 (10.8%)] have an extreme fear of COVID-19 infection. The mean score (*SD*) of fear of COVID-19 was 18.78 (5.58).

**TABLE 2:** Participants’ fear related to COVID-19.

Fear of COVID-19 Items	Strongly disagree Freq. (%)	Disagree Freq. (%)	Neutral Freq. (%)	Agree Freq. (%)	Strongly agree Freq. (%)
I am afraid of coronavirus-19	12 (6.49)	39 (21.09)	40 (21.62)	80 (43.25)	14 (7.57)
It makes me uncomfortable to think about coronavirus-19	16 (8.65)	46 (24.87)	50 (27.02)	67 (36.21)	06 (3.25)
My hands become cold and clammy when I think about coronavirus-19	34 (18.38)	80 (43.25)	35 (18.91)	33 (17.84)	03 (1.63)
I am afraid of losing my life because of coronavirus-19	29 (15.68)	66 (35.68)	39 (21.08)	42 (22.70)	09 (4.87)
When I watch news and stories about Corona Virus, I become nervous or anxious	15 (8.10)	44 (23.79)	42 (22.70)	73 (39.45)	11 (5.95)
I cannot sleep because I’m worried about getting COVID-19	52 (28.10)	92 (49.73)	27 (14.60)	13 (7.03)	01 (0.55)
My heart races or palpitates when I think about getting COVID-19	41 (22.17)	77 (41.63)	33 (17.84)	31 (16.76)	03 (1.63)
**Mean (SD) of total score: Fear of COVID-19**	**18.78 (5.58)**				
**Level of fear related to COVID-19**	**Mild fear score (7–15)**	**Moderate fear score (16–25)**		**Extreme fear score (26–35)**	
	52 (28.1)	113 (61.1)		20 (10.8)	

*N = 185.*

Table 3 presents the challenges faced by participants due to COVID-19, with the majority of study participants [120 (64.9%)] having faced moderate challenges due to the COVID-19 pandemic, one-third of them [50 (27%)] having faced high challenges, and few study participants [15 (8.1%)] having faced no challenges when posted in hospital due to the COVID-19 pandemic. The mean score (*SD*) of challenges faced due to COVID-19 was 21.39 (3.6).

**TABLE 3:** Participants’ challenges faced due to COVID-19.

Challenges faced due to COVID-19 statements	Never Freq. (%)	Sometimes Freq. (%)	Always Freq. (%)
Problem in carrying out assessment and care of patients because of constant fear of infection	32 (17.3)	102 (55.1)	51 (27.6)
Difficulty in continue handling of personal protective equipment (PPE)	10 (5.4)	37 (20.0)	138 (74.6)
Hindrance in interaction and communication because of facemask/PPE	15 (8.1)	107 (57.8)	63 (34.1)
Skin irritation/problem because of frequent use of hand rub and hand washing	32 (17.3)	106 (57.3)	47 (25.4)
Issues in handling of patients’ records and clinical assignments due to fear of contracting infection	31 (16.8)	105 (56.8)	49 (26.5)
Dealing with continue stress, fear and mental exhaustion due to COVID-19 pandemic	67 (36.2)	91 (49.2)	27 (14.6)
Ineffective clinical skill learning due to fear of COVID- 19 infection and limited clinical exposure	27 (14.6)	109 (58.9)	49 (26.5)
Lack of effective clinical supervision by teachers due to fear of contracting infection	83 (44.9)	79 (42.7)	23 (12.4)
Difficulty in completing clinical assignments like case study, case presentation and nursing care plan	16 (8.6)	62 (33.5)	107 (57.8)
Problem in counseling patients and family due to fear of infection	32 (17.3)	104 (56.2)	49 (26.5)
**Mean (SD) of total score: Challenges faced due to COVID-19**	**21.39 (3.6)**		
**Level of challenges faced due to COVID-19**	**Low challenges score (10–16)**	**Moderate challenges score (17–23)**	**High challenges score (24–30)**
	15 (8.1)	120 (64.9)	50 (27.0)

*N = 185.*

Table 4 depicts the association of fear and challenges due to COVID-19 with demographic variables among the study participants, that is, age, batch, religion, history of infection with COVID-19, history of family member infection with COVID-19, duration of clinical posting during COVID-19 pandemic. It was found that fear due to COVID-19 is not significantly associated with these demographic variables, while age, batch, and duration of clinical posting were found to be significantly associated with the challenges faced due to COVID-19 (*p*-value = 0.01).

**TABLE 4:** Independent *t*-test and ANOVA for association of fear and challenges due to COVID-19 with demographic variables among participants.

Demographic characteristics	Fear of COVID-19			Challenges faced due to COVID-19		
	Mean ± SD	*t*-value/*F*-value	*p*-value	Mean ± SD	*t*-value/*F*-value	*p*-value
**Age**						
≤20 Years	18.32 ± 4.37	0.921	0.35	20.46 ± 3.77	2.255*	0.01*
≥21 Years	19.04 ± 6.14			21.90 ± 3.42		
**Batch**						
Fresher (1st Year)	18.04 ± 4.25	1.25	0.28	20.08 ± 3.63	4.34*	0.01*
Intermediate (2nd and 3rd Year)	18.64 ± 6.05			21.65 ± 3.72		
Interns (4th Year)	19.86 ± 5.65			22.15 ± 2.98		
**Religion**						
Hindu	18.82 ± 5.63	1.414	0.22	21.38 ± 3.62	0.386	0.72
Others (Muslim/Sikh)	17.25 ± 2.06			22.00 ± 3.16		
**Did you ever had history of COVID-19 infection?**						
Yes	18.87 ± 5.67	0.742	0.46	21.38 ± 3.68	0.162	0.87
No	18.00 ± 4.63			21.50 ± 2.81		
**Did any of your family member had COVID-19 infection?**						
Yes	18.74 ± 5.50	0.377	0.71	21.41 ± 3.62	0.383	0.71
No	19.60 ± 7.07			21.00 ± 3.33		
**Duration of clinical posting during COVID-19 pandemic**						
≤2 Week	18.43 ± 5.42	1.329	0.18	20.97 ± 3.56	2.532*	0.01*
≥3 Week	19.67 ± 5.90			22.43 ± 3.52		

*N = 185.*

*Significant (p < 0.05).

## Discussion

The primary purpose of this study was to find out the fear and challenges of nursing students being in hospital for clinical posting during the COVID-19 pandemic. In this study, we found that a significant number of study participants, 113 (61.1%), had moderate fear of COVID-19 infection, similar findings have been reported for other studies where it was determined that 68.1% of students were worried about being infected with COVID-19 (Aslan and Pekince, 2021).

Another consideration is the finding of Huang et al. (2020) who found that nurses at the frontline exhibit stronger anxiety, fear, sadness, and anger than nursing students. In one study, four predictors for the fear of the coronavirus, i.e., health anxiety, regular media use, social media use, and risks for loved ones, were reported, which are similar to the findings of the present study, and 84 (45.4%) participants of that study agreed to becoming nervous or anxious when they watched news and stories about COVID-19 (Mertens et al., 2020).

De Los Santos et al. (2021) revealed in their study results that there is a moderate to high fear of COVID-19 among students across all year levels. Notably, the first-year students displayed the greatest fear among the group (*M* = 21.47, *SD* = 6.49). It was also revealed that fear of COVID-19 negatively affects the students’ sleep quality (β = −0.124, *p* = 0.045) (De Los Santos et al., 2021). In the present study, the majority of the study participants, 120 (64.9%), faced challenges due to the COVID-19 pandemic, which was replicated in a similar study, which found that longstanding challenges have emerged with the pandemic, and the processes of acceleration, change, and paralysis have marked education in these times (Lira et al., 2020).

The findings of the present study are also similar to a study conducted in China, which found that healthcare providers were challenged by working in a totally new context, exhaustion due to heavy workloads and protective gear, fear of becoming infected and infecting others, feeling powerless to handle patients’ conditions, and managing relationships in this stressful situation (Liu et al., 2020).

Our study findings which explicate that increasingly watching news and stories about COVID-19 is related to fear of COVID-19 is replicated in a similar study in which they found that more media exposure is related to more fear (Van den Bulck and Custers, 2009; Garfin et al., 2020). Galehdar et al. (2020) revealed the fear and challenges of nursing students through their qualitative data analysis, that is, death anxiety, anxiety due to the nature of the disease, fear of infecting the family, distress about wastingtime, the emotional distress of delivering bad news, fear of being contaminated, the emergence of obsessive thoughts, the bad feeling of wearing personal protective equipment, which is similar to the finding of the current study, that is, 138 (74.6%) participant faced challenges due to difficulty in continue handling of personal protective equipment (PPE), and participants [84 (45.4%)] becoming nervous or anxious when they watch news and stories about COVID-19.

## Study Limitations

This study has several limitations. Generalizability issues are also raised because of the limited sample of participants included. The study was limited to one institution. The study was limited only to female students, and data were collected after 2–4 weeks of clinical posting during the COVID-19 pandemic. Data were collected through a web survey.

## Recommendations

Future researchers are encouraged to gather a bigger sample size to derive more generalized findings. A qualitative assessment of fear and challenges faced by nursing students during the COVID-19 pandemic and its implication on their mental health may be useful to augment and better describe their lived experience with the COVID-19 pandemic.

## Conclusion

This study revealed that a significant number of nursing students had moderate fear and faced challenges due to the COVID-19 pandemic while being in hospital for clinical posting. The demographic variables such as age, batch, and duration of clinical posting were found to be significantly associated with the challenges faced due to COVID-19. COVID-19 is not going to end soon as per the existing scenario, so we require innovative stratagems or plans for nursing students in this critical time, where fear and challenges faced by students can be prevented.

## Data Availability Statement

The original contributions presented in the study are included in the article/supplementary material, further inquiries can be directed to the corresponding author.

## Ethics Statement

The studies involving human participants were reviewed and approved by Institutional Ethical Committee AIIMS Jodhpur. Undergraduate students and who have consented to participate were included in the study. The patients/participants provided their written informed consent to participate in this study.

## Author Contributions

NR considered and designed the study, conducted research, provided research materials, and collected and organized data. SS analyzed and interpreted data and did the final editing. NK wrote the initial and final draft of the manuscript and provided logistic support. All authors have critically reviewed and approved the final draft and are responsible for the content and similarity index of the manuscript.

## Conflict of Interest

The authors declare that the research was conducted in the absence of any commercial or financial relationships that could be construed as a potential conflict of interest.

## Publisher’s Note

All claims expressed in this article are solely those of the authors and do not necessarily represent those of their affiliated organizations, or those of the publisher, the editors and the reviewers. Any product that may be evaluated in this article, or claim that may be made by its manufacturer, is not guaranteed or endorsed by the publisher.
